# Trait‐mediated indirect interactions: Moose browsing increases sawfly fecundity through plant‐induced responses

**DOI:** 10.1002/ece3.5581

**Published:** 2019-08-23

**Authors:** Michelle Nordkvist, Maartje J. Klapwijk, Lars Edenius, Jonathan Gershenzon, Axel Schmidt, Christer Björkman

**Affiliations:** ^1^ Department of Ecology Swedish University of Agricultural Sciences Uppsala Sweden; ^2^ Department of Wildlife, Fish, and Environmental Studies Swedish University of Agricultural Sciences Umeå Sweden; ^3^ Department of Biochemistry Max Planck Institute for Chemical Ecology Jena Germany

**Keywords:** di‐terpenoid resin acids, herbivore–herbivore interactions, host plant quality, lateral interactions, plant–herbivore interactions, trophic interaction modifications

## Abstract

1. Induced responses in plants, initiated by herbivory, create potential for trait‐mediated indirect interactions among herbivores. Responses to an initial herbivore may change a number of plant traits that subsequently alter ecological processes with additional herbivores. Although common, indirect interactions between taxonomically distant herbivores, such as mammals and insects, are less studied than between taxonomically related species (i.e., insect–insect). In terms of mammal–insect interactions, effects on insect numbers (e.g., density) are relatively well studied, whereas effects on performance (e.g., fecundity) are rarely explored. Moreover, few studies have explored mammal–insect interactions on coniferous plants.
2. The aim of this study was to investigate the effect of mammalian induced responses on insect performance. We specifically investigated the effect of moose (*Alces alces*) browsing on Scots pine (*Pinus sylvestris*) and subsequent effects on sawfly (*Neodiprion sertifer*) performance.
3. Sawfly larvae were reared on browsed, clipped, and unbrowsed control pine trees in a controlled field experiment. Afterward, cocoon weight was measured. Needle C:N ratio and di‐terpene content were measured in response to browsing.
4. Sawfly performance was enhanced on trees browsed by moose. Cocoon weight (proxy for fecundity) was 9 and 13% higher on browsed and clipped trees compared to unbrowsed trees. Cocoon weight was weakly related to needle C:N ratio, and browsed trees had lower a C:N ratio compared to unbrowsed trees. Needle di‐terpene content, known to affect sawfly performance, was neither affected by the browsing treatments nor did it correlate with sawfly weight.
5. We conclude that mammalian herbivory can affect insect herbivore performance, with potential consequences for ecological communities and with particular importance for insect population dynamics. The measured plant variables could not fully explain the effect on sawfly performance providing a starting point for the consideration of additional plant responses induced by mammalian browsing affecting insect performance.

## Graphical abstract

The aim of this study was to investigate the effect of mammalian induced responses on insect performance. We specifically investigated the effect of moose browsing on Scots pine trees and subsequent effects on sawfly performance. Sawfly performance was enhanced on trees browsed by moose. Cocoon weight (proxy for fecundity) was 9 and 13% higher on browsed and clipped trees compared to unbrowsed trees. We conclude that mammalian herbivory can affect insect herbivore performance.

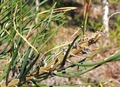

## 1 INTRODUCTION

Trait‐mediated indirect interactions (defined by Abrams, 1995) are abundant in ecological systems, affecting various processes and interactions with implications for individual performance, population fluctuations, and community composition (Van Veen, Van Holland, & Godfray, 2005; Werner & Peacor, 2003). Their importance has received increasing attention, both theoretically (Golubski & Abrams, 2011; Terry, Morris, & Bonsall, 2017) and empirically (Ando, Utsumi, & Ohgushi, 2017; Nakamura, Miyamoto, & Ohgushi, 2003; Soler et al., 2012), but their effects are not yet fully understood. Trait‐mediated indirect interactions (from now “indirect interactions”) are particularly common in plant–herbivore communities, as plants are subjected to herbivory by several species of herbivores often without lethal consequences. Trait‐mediated effects can link several levels in an ecological community that would not directly interact and could potentially have a large effect on the species involved, changing both top‐down, lateral, and bottom‐up processes (Erwin, Züst, Ali, & Agrawal, 2014; Muiruri, Milligan, Morath, & Koricheva, 2015; Ohgushi, 2005; Terry et al., 2017). Studies in insect–insect systems have shown that the specificity of the herbivores is vital to the outcome of the indirect interaction. Different species might initiate different responses in one plant species, and the same plant response might generate different effects on different receiver herbivores (Agrawal, 2000).

Since indirect interactions are prevalent in terrestrial plant–herbivore systems, we conducted a systematic literature search to gain an overview of studied interactions (Table 1, Method S1 in Appendix S1). We found that previous research has focused mainly on indirect interactions between taxonomically similar species (cf. Ohgushi, 2005), like insect herbivores, whereas interactions between taxonomically more distant species, such as mammals and insects, have been less emphasized. In addition, when studying indirect effects between mammals and insects, most studies focus on effects on density, species richness, or inflicted feeding damage. In terms of the mediating plant species, studies have been conducted on either herbs or deciduous trees. Far less investigated are (a) the effect of mammalian herbivory on insect herbivore performance such as survival and fecundity, (b) the mechanisms underlying these trait‐mediated indirect effects, and (c) mammal–insect indirect interactions on coniferous plants (Table 1). Our study aims to fill these gaps in knowledge by studying an ungulate—conifer—insect herbivore system to explore tree response to browsing, changes in foliage quality and measure insect performance. Deciduous and coniferous trees are known to respond differently to browsing by the same mammalian herbivore (Danell, Bergström, & Edenius, 1994; Stolter, Ball, Julkunen‐Tiitto, Lieberei, & Ganzhorn, 2005), providing an additional incentive for this study.

**Table 1:** Papers on trait‐mediated indirect interactions between mammals and insects

Paper	System	Type of response	Natural or controlled addition of herbivores, study conducted in laboratory or field	Plant trait(s) measured	Main result and direction of effect
Tabuchi, Ueda, and Ozaki (2010)	Sika deer (*Cervus nippon*) Dwarf bamboo (*Sasa nipponica*) Gall midge (*Procystiphora uedai*)	Performance and behavior	Natural, field	Shoot size and softness	+ neonate survival − ovipositing − larval and pupal weight
Lind, Myron, Giaccai, and Parker (2012)	White‐tailed deer (*Odocoileus virginianus*) Spicebush (*Lindera benzoin*) Foliar insect herbivores (unspecified, field) & Spicebush swallowtail (*Papilio troilus*, laboratory)	Performance (laboratory) and damage (filed)	Natural, field – Controlled, laboratory	− nitrogen, +carbon, + water content, + SLA	Field (all herbivory): − damage Laboratory (only specialist swallowtail): + preference, + growth
Simonsen and Stinchcombe (2007)	Clipping Ivyleaf morning glory (*Ipomoea hederacea*) *Spodoptera exigua*	Performance and damage	Controlled, laboratory		No effect
Martinsen et al. (1998)	Beaver (*Castor canadiensis*) Cottonwood (*Populus* fremontii x P. angustifolium) Chrysomela confluens	Performance and numerical	Natural, field Controlled, laboratory	+ resprout growth, + phenolic glycosides, + nitrogen	+ density, + defense ability, + adult mass, + larval period
Hrabar and Du Toit (2014)	Elephant (*Loxodonta africana*) Mopane trees (*Colophospermum mopane*) Mopane moths (*Imbrasia belina*)	Behavior	Natural, field	− tannin:protein ratio (measured plant trait did not relate to ovipositing preference, suggested trait responsible for effect: available biomass)	− ovipositing
Takagi and Miyashita (2012)	Sika deer (*Cervus nippon*) Woody vine (*Aristolochia kaempferi*) Swallowtail butterfly (*Byasa alcinous*)	Behavior	Natural, field	+ % young leaves (regrowth), +nutrients	+ ovipositing
Moe, Gjorvad, Eldgards, and Hegland (2018)	Red deer (*Cervus elaphus*) Bilberry (*Vaccinium myrtillus*) Folivorous larvae (mainly Lepidoptera)	Behavior and damage	Controlled, laboratory		Light browsing: + damage and preference High browsing: − damage & preference
Bultman et al. (2018)	Sheep (*Ovis aries*) Perennial rye grass (*Lolium perenne*) Argentine stem weevil (*Listronotus bonariensis*)	Damage	Controlled, laboratory		− damage
Kellner and Swihart (2017)	White‐tailed deer (*Odocoileus virginianus*) & Eastern cotton tailed rabbits (*Sylvilagus floridanus*) White oak (*Quercus alba*) & black oak (*Quercus velutina*) Foliar insect herbivores (unspecified)	Damage	Natural		+ damage
Muiruri et al. (2015)	Moose (*Alces alces*) Silver birch (*Betula pendula*) Foliar insect herbivores (unspecified)	Damage	Natural		+/− damage depending on associational effects
Schwenk and Strong (2011)	Moose (*Alces alces*) Striped maple (*Acer pensylvaticum*) Foliar insect herbivores (unspecified)	Damage	Natural		+ damage
Olofsson, Dahlgren, and Witzell (2007)	Gray‐sided vole (*Clethrionomys rufocanus*) Northern willow (*Salix glauca*) Foliar invertebrate herbivores (Lepidoptera, Hymenoptera, slugs)	Damage	Natural	+ leaf size, + nutrients, + number of leafs	+ damage
Den Herder, Bergström, Niemelä, Danell, and Lindgren (2009)	Moose (*Alces alces*) Silver birch (*Betula pendula*) Foliar insect herbivores	Damage and numerical	Natural		Summer browsing: − damage Summer and winter browsing: + abundance of aphids No effect on leaf miners or weevils
Ostrow, Huntly, and Inouye (2002)	Pocket gopher (*Thomomys talpoides*) *Medicago sativa, Medicago officinalies* (mixed) Various herbivorous insects	Damage and numerical	Natural		− density of sucking insects +damage of chewing insects
Takagi and Miyashita (2015)	Sika deer (*Cervus nippon*) Woody vine (*Aristolochia kaempferi*) Swallowtail butterfly (*Byasa alcinous*)	Numerical	Natural	+proportion of young leafs	+abundance
Bailey and Whitham (2006)	Beaver (*Castor canadiensis*) Cottonwood (*Populus angustifolia*) Galling sawfly (*Phyllocolpa* sp.)	Numerical	Natural	+shoot length	+abundance
Bailey and Whitham (2003)	Elk (*Cervus canadensis*) Aspen (*Populus tremula*) *Phyllocalpa bozemanii*	Numerical	Natural		− number
Bailey and Whitham (2002)	Elk (*Cervus canadensis*) Aspen (*Populus tremula*) Various leaf chewers	Numerical	Natural		+ richness, + abundance
Olofsson and Strengbom (2003)	Reindeer (*Rangiferus tarandus*) *Salix lanata* *Pontania glabrifons*	Numerical	Natural		+ density
Danell and Huss‐Danell (1985)	Moose (*Alces alces*) Birch (*Betula pendula, B. pubescens*) *Symydobius oblogus*, *Psylla betulae*	Numerical	Natural	+ leaf size, + nitrogen, + chlorophyll	+ density
Roininen, Price, and Bryant (1997)	Snowshoe hare (*Lepus americanus*), Moose (*Alces alces*) Cottonwood (*Populus balsamifer*) *Phyllocalpa* spp.	Numerical	Natural	+ shoot length and vigor	+ number
Roininen et al. (1997)	Snowshoe hare (*Lepus americanus*), Moose (*Alces alces*) *Salix novaeangliae* *Phyllocalpa* spp.	Numerical	Natural	+ shoot length and vigor	+ number
Hjältén and Price (1996)	Eastern cottontail rabbit (*Sylvilagus floridanus*) *Salix lasiolepis* Galling sawfly (*Euura lasiolepis*)	Numerical	Natural	+ shoot length	+ density
Gómez and González‐Megías (2002)	Sheep (*Ovis* sp.), Ibex (*Capra pyrenaica*) *Hormathophylla spinosa* *Timarcha lugens*	Numerical	Natural	− flower number, − fruit abundance	− density

Note

First column states the author(s) and year of publication, and the following columns describe features of the studies (Column 1—study system; Column 2—type of insect response: performance, behavior, and/or damage; numerical: abundance, density, richness, or diversity; Column 3—experimental setup: natural or controlled; Column 4—measured plant traits; when blank, no plant trait has been measured; Column 5—and main result). Papers published before 2005 are reviewed in Ohgushi (2005). The remaining papers were identified through two Web of Science literature searches (search method provided in Appendix S1, Method S1).

Available nitrogen commonly affects the performance of herbivorous insects (Mattson, 1980), and increased plant nitrogen often increases herbivore performance (e.g., Awmack & Leather, 2002; Joern & Behmer, 1997). Additionally, the level of plant defense often affects insect performance (Awmack & Leather, 2002). Consequently, if herbivory induces changes to either of these plant traits, it could affect the performance of a subsequent herbivore (Ali & Agrawal, 2014). Plant responses to mammalian herbivory with respect to these traits are varied. Studies show both increased and decreased nutritional quality (Nykänen & Koricheva, 2004 and references therein) and levels of chemical defenses (Bryant, Chapin, & Klein, 1983; Bryant, Wieland, Clausen, & Kuropat, 1985). Changes in traits that determine plant quality are often considered the mechanism underlying observed patterns of indirect interactions between mammals and insects (Table 1). But studies on insect herbivore performance in response to mammalian browsing damage rarely link the measured induced plant responses to insect performance by testing for a relationship between plant response and insect performance (but cf. Martinsen, Driebe, & Whitham, 1998).

**Figure 1:**
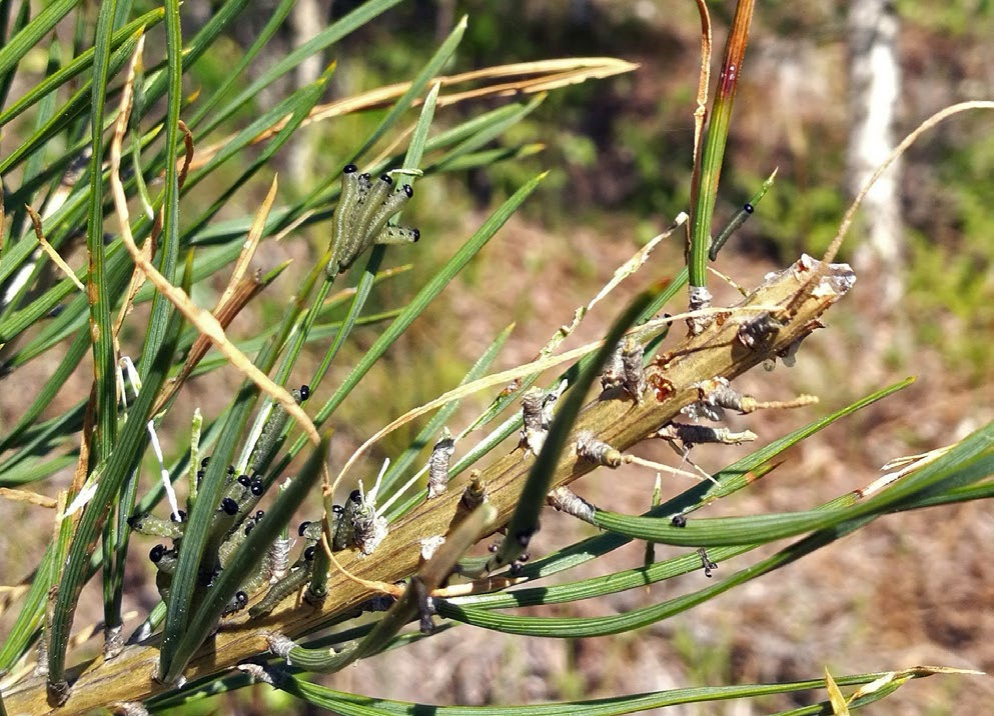
European pine sawflies (*Neodiprion setifer*) larvae feeding on a Scots pine (*Pinus sylvestris*) branch previously browsed by moose (*Alces alces*)

We aim to investigate induced responses by mammalian browsers on plant chemistry, the effect of plant chemistry on insect performance, and the link between changes in plant chemistry and insect response. In order to do so, we use a controlled field experiment to examine the indirect interaction between ungulates and the performance of herbivorous insects. Controlled experiments, opposite to observational studies, are preferable when the aim is to disentangle potential mechanisms. Our study system consists of a specialist herbivore, the European pine sawfly (*Neodiprion sertifer*), and ungulate browsers [primarily moose(*Alces alces*)], both feeding on Scots pine (*Pinus sylvestris*) but at different periods of the growth season. Separately, the effect of moose browsing on plant traits (e.g., Edenius, Danell, & Bergström, 1993; Nykänen & Koricheva, 2004) and the effects of pine traits on sawfly performance (Björkman, Larsson, & Bommarco, 1997; Björkman, Larsson, & Gref, 1991; Larsson, Björkman, & Gref, 1986; Niemelä, Tuomi, & Lojander, 1991) have been extensively studied (a summary of previous results can be found in Appendix S1, Table S1 in Appendix S1). The novelty of our study is that we experimentally examine the chain of effects from browsing to pine traits to insect performance and explore moose browsing effects on insect‐specific pine traits. The benefits of using this particular study system are, first, that sawflies feed exclusively on previous years’ needles, allowing us to study trait‐mediated interaction through chemical responses in existing foliage. Second, sawflies are easy to move as eggs/young larvae allowing us to add sawflies to pines, controlling their densities and avoiding biases in the response created by potentially different insect herbivore densities. Third, it allows us to study indirect interactions on a coniferous plant species. And fourth, we know from observations in the field that sawfly females do not avoid pine trees with browsing damage (cf. Figure 1). Based on previous studies, we expected that the effects of winter browsing would lead to increased nutritional quality of the pines (Nykänen & Koricheva, 2004). In addition, we expected the reduction of photosynthetically active tissue to cause lower levels of carbon‐based defenses, such as terpenes (Bryant et al., 1983; Du Toit, Bryant, & Frisby, 1990). These changes should have a positive effect on sawfly performance as previous work indicates that sawflies respond positively to high nutrients and low di‐terpene levels (Björkman et al., 1997, 1991). In order to achieve our goal, we measure the effect of browsing on (a) weight and egg load of sawfly females, (b) plant quality, both in terms of nutrients and chemical defenses, and (c) investigate whether induced changes in plant quality could be the underlying mechanism through which browsing affects insect herbivore performance.

## 2 MATERIALS AND METHODS

### 2.1 Study species

The European pine sawfly (*N. sertifer* Geoffr.; Hymenoptera, Diprionidae) is a folivorous insect, specializing on *Pinus* spp.. Eggs are laid during late August–early September on several needles in batches of 50–120 eggs on current year's needles. Larvae hatch in spring and feed gregariously during early summer. After hatching, the larvae continue to feed on the needles in which the eggs were laid, preferably avoiding the needles from the newly developing shoots.

Scots pine is a coniferous tree species native to Sweden and Eurasia and one of the dominating tree species in the forests in Sweden. Terpenes and phenolics are some of the most important defense compounds in pines (Mumm & Hilker, 2006).

Moose is widely distributed over Sweden with local variations in density. In winter time, a main food source is pine shoots (Bergström & Hjeljord, 1987). Moose winter browsing can have profound effects on Scots pine growth and morphology (Edenius et al., 1993; Wallgren, Bergquist, Bergström, & Eriksson, 2014), and likely increases nutritional quality, as shown in similar interactions (Nykänen & Koricheva, 2004).

### 2.2 Experimental design

The experiment was set up in 2016 at two field sites in semi‐natural young forest stands near Uppsala, south‐central Sweden (Site 1:59 52 01.7N, 18 11 06.4E, Site 2:50 58 00.9N, 18 13 37.0E), with Scots pine as the dominant tree species. At each site, eight blocks were set up so that all trees within a block were growing in similar conditions to minimize potential effects of within‐site variation in microclimates. In each block, six trees were selected. Two of the six trees were selected because they had been previously browsed. The additional four trees were unbrowsed. These four trees were randomly assigned a treatment, control or clipping. The average height of the selected trees was 174 cm (range 107–241 cm).

Based on the nature of the damage and knowledge about local ungulate populations in our sites, most browsing damage can be attributed to winter browsing by moose. The range of browsing intensity on naturally browsed trees within our sites ranged between 25% and 75% with an average of 50% of the lateral shoots browsed (trees with leader shoot browsed were not included). We made sure that the browsing on the selected browsed trees was fresh and thus occurred in the current winter, trees with clear signs of older damage were avoided. We simulated browsing by clipping lateral shoots resembling 50% browsing damage. Clipping is a commonly used method to mimic ungulate browsing, and plant growth responses to clipping are similar to responses to browsing (Edenius, 1993; Wallgren et al., 2014). To simulate winter browsing, the trees were clipped while still in dormancy, early spring 2016. Using the clipping treatment alongside, the browsing treatment allowed us to “confirm” that the differences in plant quality were browsing‐induced and not an effect of selective browsing on, for example, trees with low defense levels (Stolter et al., 2005). To summarize, our experiment was replicated in two sites that each contained eight blocks with six trees, two naturally browsed, two clipped, and two control trees.

### 2.3 Needle chemistry

Needle samples were collected from all trees after the clipping treatment but prior to sawfly exposure. We made sure to collect the needles from the same whorl where we would later add the sawfly larvae. Since sawfly larvae feed exclusively on foliage from the previous year and remain on the branch on which they hatch or are placed, we consider this the appropriate way of sampling needles for studying plant systematic response to browsing and effects on sawflies. Samples were instantly frozen using dry ice (in the field) and subsequently stored in −22°C prior to analyses. Needles for analysis of carbon and nitrogen content were first dried (70°C for 48 hr) and then ground. Total carbon and nitrogen content (% dry weight) was analyzed with an elemental analyzer:vario EL CNS (Elementar Analysensysteme GmbH, Elementar‐Strasse 1, D‐63505, Langenselbold, Germany). Gas chromatography–mass spectrometry (GC‐MS) was used to analyze needles for di‐terpenoids. Firstly, needles were ground in an oscillating mill (Retsch MM400) using liquid nitrogen during the grinding process to keep the needles frozen. A total of 100 mg of needles were then extracted in 1 ml of tert‐butyl methyl ether [including an internal standard of di‐chlorodehydroabietic acid (50 µg/ml)] (Cansyn, Canada) and shaken for 14 hr. Ethereal extracts were then washed with 0.3 ml of 0.1 M ammonium carbonate (NH_4_)_2_CO_3_ (pH 8.0) and subsequently transferred into new vials. A total of 50 µl of 0.2 M N‐tri‐methylsulfoniumhydroxid (Macherey‐Nagel, Germany) was added to the ethereal extracts to methylate the di‐terpenoids, and samples were incubated at room temperature for 1 hr. After centrifugation at 4,000 *g* for 5 min, supernatants were transferred into new vials. Analyses of the derivatized samples for di‐terpenoid compounds were performed on a Hewlett‐Packard 6890 GC‐MSD system connected to an Agilent 5973 Network Mass Selective Detector and a Zebron ZB‐5 MSi column (30 m × 0.25 mm × 0.25 μm) (Phenomenex, Germany). Injections were made with 1 µl of ethereal extract. GC‐MS split ratios were 1:10 with an injector temperature of 220°C. Column operating temperature was set to 150°C during the three first minutes of the program and then subsequently increased with 3.5°C/min up to 280°C. The final temperature was held for four minutes. Helium was used as carrier gas, with a constant flow rate of 1 ml per minute. Di‐terpenoid compounds were identified by comparing the retention times and mass spectra from authentic standards or mass spectra in the Wiley 275.L or National Institute of Standards and Technology 98.1 MS libraries. Di‐terpenoid content was quantified in relation to the internal standard. Seven di‐terpenes were determined in the needles (manoyl oxide, sandaracopimaric acid, levopimaric acid, dehydroabietic acid, abietic acid, neoabietic acid, pinifolate). We summed the contents to obtain total di‐terpenoid content. Carbon and nitrogen content were analyzed for all trees (*n*_control_ = 32, *n*_browsed_ = 32, *n*_clipped_ = 32), whereas di‐terpene content was analyzed only on the trees that were included in the sawfly treatment, with the exception of six trees that were excluded due to difficulties arising while running the di‐terpene analysis (*n*_control_ = 9, *n*_browsed_ = 10, *n*_clipped_ = 9). Both total di‐terpene content and individual compound content were analyzed with respect to the treatment.

### 2.4 Collection and preparation of sawfly larvae

Sawfly larvae were collected in May 2016 from an outbreak area near Oskarshamn, coastal‐southern Sweden (57 8 42.4N, 16 17 55.3E). Larvae were stored for three days in 5°C dark room, until reaching 2nd instar, and then randomly assigned to groups (mean ± standard deviation group size: 51.7 ± 7.7) to avoid maternal effects. We are aware that previous research has shown that di‐terpenes have the largest effect on sawfly larvae during early instars via increased larval mortality (Larsson et al., 1986). As our main aim was to study fecundity and not survival, we concentrated on getting the larvae well‐established rather than quantifying effects in the 1st instar, hence placing them on the trees early on in their 2nd instar. Still, it is possible that effects of plant quality on fecundity are operating also during the 1st instar, which was then not picked up by our method. Larval groups were placed on the trees on the 19th of May 2016 and caged in mesh bags to exclude predation. Three larval groups were placed out per block, randomly placed on a control, a clipped, and a browsed tree. This controlled addition of herbivores, rather than natural colonization, eliminates the potential bias that higher quality plants are colonized by already higher performing individuals and/or that density of herbivores influences their performance. Larval groups were left to feed throughout all their larval instars until cocoon spinning. The cocoons were collected from the field and brought to the laboratory.

### 2.5 Performance measurements

All cocoons were counted, weighed, and based on weight determined as female or male. There is a distinct size difference between female and male sawflies, females being larger than males (Kolomiets, Stadnitskii, & Vorontsov, 1979). Female cocoon weight was used as a measure of performance. Cocoon weight is a well‐established proxy for fecundity in diprionid sawflies (Heliövaara, Väisänen, & Varama, 1990; Raffa, Krause, & Reich, 1998). All cocoons were reared outside in separate vials and sheltered from predation and precipitation in ambient conditions. By rearing out the sawflies, we could confirm the determination of females/males. We then added the females wrongly determined as males to the data set. A randomly selected subsample, from each group per tree per block of the emerged females, was dissected, and body weight, abdomen weight, and number of eggs were measured (*n*_control_ = 42, *n*_browsed_ = 43, *n*_clipped_ = 47). This is a way to confirm that higher pupal weight is translated into a larger number of eggs.

### 2.6 Statistical analysis

All data were analyzed in R software version 3.2.4 (R Core Team, 2016) using the lme function in the nlme package (Pinheiro, Bates, DebRoy, & Sarkar, 2016) to fit linear mixed effects models and the glmer function in the lme4 package (Bates, Maechler, Bolker, & Walker, 2015) to fit generalized mixed effects models. To calculate the model results, we used the car package (Fox & Weisberg, 2011). Assumptions for normality, homogeneity, and independence were checked by inspecting residuals and using Levene's test for homogeneity of variance (leveneTest; R‐package car).

#### 2.6.1 Insect performance

Differences in cocoon and body weight were analyzed using mixed effects linear models with browsing treatment as a fixed factor. To account for variability within each tree in a block within a site, we used tree identity nested in block nested in site as a hierarchical random factor. The response variable cocoon weight showed unequal variances between the different treatments, and this required us to use a variance structure that would allow for different variance spreads per treatment. The variance structure incorporates the pattern of unequal variances into the model and takes it into account in the analysis (varIdent; car package). Differences in number of eggs were analyzed using generalized linear mixed model with Poisson distribution, with browsing treatment as a fixed factor and again the hierarchical structure for site, block, and tree as random factor.

#### 2.6.2 Plant quality

C:N ratio, nitrogen content, carbon content, and di‐terpene content were analyzed using mixed effects linear models with browsing treatment as a fixed factor and block identity nested in site as a random factor.

#### 2.6.3 Relationship between plant quality and insect performance

To test whether sawfly performance was related to plant quality, we performed regression analyses assessing the relationship between plant C:N ratio as well as di‐terpene content and sawfly cocoon weight. To assess whether a higher weight corresponded to a larger number of eggs, we performed regression analyses for number of eggs with cocoon weight, body weight, and abdomen weight. We also analyzed whether the browsing treatments affected sawfly survival, using a linear mixed effect model.

## 3 RESULTS

### 3.1 Insect performance

**Figure 2:**
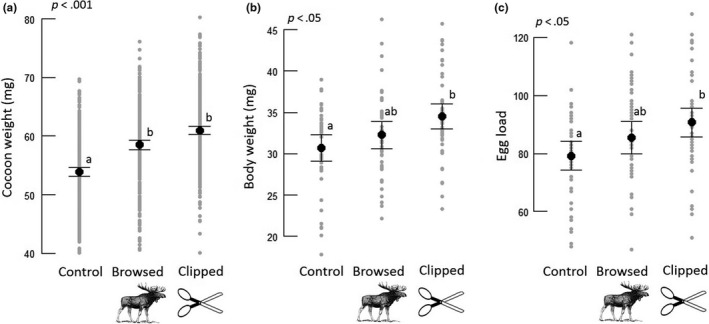
(a) Cocoon weight for female sawflies (*Neodiprion sertifer*) on control, browsed, and clipped pine trees (*n*_control_ = 301, *n*_browsed_ = 291, *n*_clipped_ = 339). Mean weights were 53.9, 58.5, and 61.0 mg for control, browsed, and clipped trees, respectively. (b) Body weight (*n*_control_ = 42, *n*_browsed_ = 43, *n*_clipped_ = 47), mean weight 30.7, 32.3, and 34.5 for control, browsed, and clipped trees and (c) number of eggs (*n*_control_ = 42, *n*_browsed_ = 43, *n*_clipped_ = 47), mean number of eggs 79, 86, and 91 for control, browsed, and clipped trees, for female sawflies. The *p*‐values in the graphs indicate value for the whole model, and letters indicate significant differences between individual treatments. Body weight and number of eggs were measured on a subset of the females (*n*_control_ = 42, *n*_browsed_ = 43, *n*_clipped_ = 47). Black data points represent mean values, arrows represent one standard error, and gray data points are individual observations

Weight of female sawfly cocoons was 9% and 13% higher on browsed and clipped trees, compared to control trees, respectively (Figure 2a, Table 2). Female body weight was 13% higher on clipped trees, whereas there was no significant difference between browsed and control trees (Figure 2b, Table 2). Number of eggs per female was 15% higher when larvae had been reared on clipped trees compared to controls, while there was no significant difference between females that were reared as larvae on control and browsed trees (Figure 2c, Table 2). There were no significant differences between browsed and clipped trees in any of the measured sawfly traits. Survival did not differ between treatments, and mean (±*SD*) survival (from 2nd instar until pupation) for all larvae was 69% (±32%). There was no difference in male cocoon weight.

**Table 2:** ANOVA (type II test) and summary table for linear mixed effects model testing the difference in cocoon weight (mg) and body weight (mg) for female sawflies (*Neodiprion sertifer*) in relation to browsing and for the generalized linear mixed effects model testing the difference in number of eggs for female sawflies in relation to browsing

Cocoon weight (mg)					
**Fixed**	**Estimates**	***SE***	***χ*^2^**	***df***	***p*‐value**
Intercept	53.99	1.25			<.001
Browsing			14.28	2	**<.001**
Naturally browsed	4.6	1.75			*
Clipped	6.3	1.71			*
**Random**	**Intercept**	**Residuals**			
Site	0.00096				
Site/Block	0.76				
Site/Block/Tree	3.90	5.99			
**Body weight (mg)**					
**Fixed**	**Estimates**	***SE***	***χ*^2^**	***df***	***p*‐value**
Intercept	30.71	0.98			<.001
Browsing			8.81	2	**<.05**
Naturally browsed	1.51	1.34			
Clipped	3.80	1.30			*
**Random**	**Intercept**	**Residuals**			
Site	0.00029				
Site/Block	1.16				
Site/Block/Tree	1.93	4.67			
**Number of eggs**					
**Fixed**	**Estimates**	***SE***	***χ*^2^**	***df***	***p*‐value**
Intercept	4.37	0.039			<.001
Browsing			6.06	2	**<.05**
Naturally browsed	0.077	0.056			
Clipped	0.13	0.052			*
**Random**	**Intercept**	**Residuals**			
Site	0				
Site/Block	0				
Site/Block/Tree	0.015	0.12			

Note

Browsing treatment (control, browsed, and clipped) was used as fixed factor and site, block and tree as random factors. *p*‐values represent significance for the overall model, significant *p*‐values for the browsing treatment are marked in bold and asterisks (*) represent significant differences between individual browsing treatments and control treatment.

### 3.2 Relationship between number of eggs and weight

Number of eggs was positively related to cocoon weight, body weight, and abdomen weight (Appendix S1, Figure S1), confirming that higher weight is directly related to higher (potential) fecundity.

### 3.3 Plant quality

**Figure 3:**
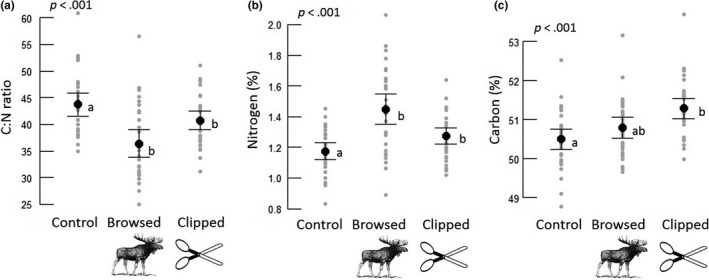
(a) C:N ratio of control, browsed, and clipped pine trees (*n*_control_ = 32, *n*_browsed_ = 32, *n*_clipped_ = 32). Mean ratios were 43.7, 36.4, and 40.8 for control, browsed, and clipped trees, respectively. (b) Nitrogen content (%) of control, browsed, and clipped pine trees (*n*_control_ = 32, *n*_browsed_ = 32, *n*_clipped_ = 32). Mean content were 1.18, 1.45, and 1.27% in control, browsed, and clipped trees, respectively. (c) Carbon content (%) of control, browsed, and clipped pine trees (*n*_control_ = 32, *n*_browsed_ = 32, *n*_clipped_ = 32). Mean content were 50.5, 50.8, and 51.3% in control, browsed, and clipped trees, respectively. Black data points represent mean values, arrows represent one standard error, and gray data points are individual observations

C:N ratio was on average 17% lower on browsed trees compared to controls and 6.6% lower on clipped trees compared to control trees (Figure 3a, Table 3). Nitrogen content was 23% higher in browsed trees compared to controls and 7.6% higher in clipped trees (Figure 3b, Table 3). Carbon content was 1.6% higher in clipped trees compared to controls, while there was no difference between browsed and control trees (Figure 3c, Table 3). Pine di‐terpene content was not affected by any of the browsing treatments.

**Table 3:** ANOVA (type II test) and summary table for linear mixed effects model testing the difference in pine C:N ratio, nitrogen content (%), and carbon content (%) in relation to browsing (*n*_trees_ = 32, 32, 32)

**C:N ratio**					
**Fixed**	**Estimates**	***SE***	***χ*^2^**	***df***	***p*‐value**
Intercept	43.72	1.21			
Browsing			31.82	2	**<.001**
Naturally browsed	−7.35	1.31			*
Clipped	−2.96	1.31			*
**Random**	**Intercept**	**Residuals**			
Site	0.0007				
Site/Block	3.10	5.25			
**Nitrogen (%)**					
**Fixed**	**Estimates**	***SE***	***χ*^2^**	***df***	***p*‐value**
Intercept	1.18	0.45			
Browsing			39.35	2	**<.001**
Naturally browsed	0.22	0.44			*
Clipped	0.10	0.44			*
**Random**	**Intercept**	**Residuals**			
Site	0.031				
Site/Block	0.098	0.18			
**Carbon (%)**					
**Fixed**	**Estimates**	***SE***	***χ*^2^**	***df***	***p*‐value**
Intercept	50.50	0.34			
Browsing			24.24	2	**<.001**
Naturally browsed	0.29	0.16			
Clipped	0.79	0.16			*
**Random**	**Intercept**	**Residuals**			
Site	0.46				
Site/Block	0.12	0.65			

Note

Browsing treatment (control, browsed, and clipped) was used as a fixed factor and site and block as random factors. *p*‐values represent significance for the overall model, significant *p*‐values for the browsing treatment are marked in bold and asterisks (*) represent significant differences between individual browsing treatments and control treatment.

### 3.4 Relationship between performance and plant quality

**Figure 4:**
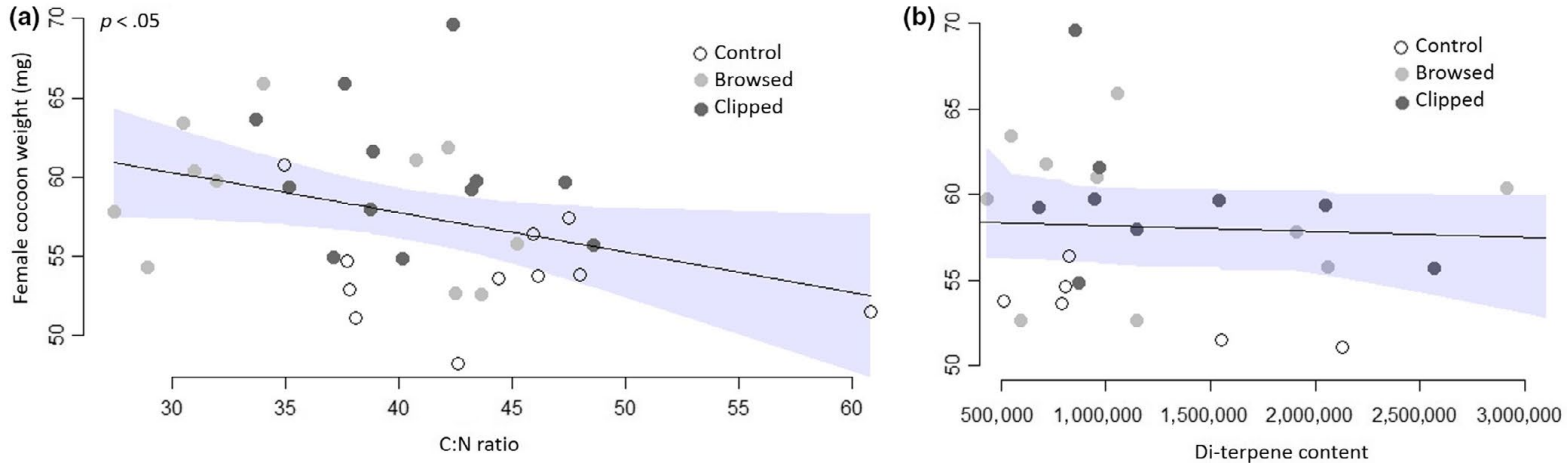
Relationship between pine sawfly (*Neodiprion sertifer*) female cocoon weight (mg) and (a) pine C:N ratio or (b) pine di‐terpene content. Shaded area represents 95% confidence interval. R‐squared is 13% for relationship between cocoon weight and C:N ratio. Data points represent pooled sawfly cocoon weights (mg) per tree. Color of the data points represents the treatment (white = control, light gray = browsed, dark gray = clipped). *p*‐value represents the result for the regression analysis, and the line represents the model fit

Sawfly cocoon weight was negatively related to C:N ratio (*p* < .05), although the variation was high resulting in low explanatory power (*R* ^2^: 13%) (Figure 4a). There was no significant relationship between number of eggs or body weight and C:N ratio. There was no significant relationship between any of the sawfly performance measures and needle di‐terpene content (Figure 4b).

## 4 DISCUSSION

Our results demonstrate that mammalian browsing can change plant traits with subsequent positive effects on insect performance. In our system, the trait‐mediated effect was manifested through higher cocoon weight of the European pine sawfly as a consequence of larval feeding on naturally browsed or clipped pine trees, compared to unbrowsed control trees. Our results demonstrate higher fecundity (eggs per female) on clipped trees and a trend for higher fecundity on browsed trees (Figure 2), which is supported both by the strong relation between number of eggs and cocoon weight (Appendix S1, Figure S1), and that cocoon weight is a well‐known proxy for fecundity (e.g., Heliövaara et al., 1990). We found that the investigated plant traits (C:N ratio) partially explained the observed effects on sawfly performance.

The unexpected weak effect of browsing on sawfly performance could be due to multiple factors, such as adding 2nd instar larvae rather than neonates or the time lag between moose and sawfly herbivory, which we will discuss further down in the discussion. The chosen method of controlled field experiment might have lowered the magnitude of effect compared to previous studies (e.g., Martinsen et al., 1998). Effect of plant responses is only one component of an overall trait‐mediated effect. Other parameters such as female oviposition choice or density could influence the outcome of the interaction, and female sawflies are known to make oviposition choices depending on plant quality (Björkman et al., 1997). Even though our method might have weakened the magnitude of the effect that we aimed to study, a 9%–13% higher fecundity could still significantly impact population dynamics (Larsson, Ekbom, & Björkman, 2000).

Previous studies have shown that mimicking browsing on pine by clipping induces similar responses in growth to natural browsing (Edenius, 1993). These growth responses, either due to clipping or browsing, most likely change nutrient allocation. Our study confirmed this, as the response in C:N ratio was lower in both browsed and clipped trees compared to control trees (Figure 3). C:N ratio was similar between browsed and clipped trees although the response was weaker in clipped trees. One potential explanation could be preferential browsing on high‐quality trees, hence that our selected browsed trees were higher in nitrogen prior to browsing, although previous research suggests that ungulates selectively feed on trees with low defense rather than of high nutritional quality (e.g., Bryant & Kuropat, 1980; Stolter et al., 2005). An alternative explanation could be the presence of intrinsic differences between browsing and clipping. One such difference could be the presence of ungulate saliva in the tree wound. Studies have shown that the addition of mammalian herbivore saliva can change the plant responses in addition to mechanical damage (Bergman, 2002; Ohse et al., 2017). Even though there might be factors additional to the mechanical damage influencing plant responses, our most important finding is the direction of the response to natural browsing and clipping is similar, in comparison with the control trees.

Based on previous research, we expected a negative relationship between C:N ratio in pine needles and the performance of *N. sertifer* (Björkman et al., 1997, 1991; Larsson et al., 2000). The results of our study confirm the direction of the effect (negative) but the relationship between sawfly cocoon weight and C:N ratio is weak (Figure 4a). Leading to the conclusion that, even though C:N ratio affects sawfly performance, it is not the sole mechanism explaining the enhanced sawfly performance on browsed trees.

We investigated the potential reduction of carbon‐based defense compounds as another possible mechanism. Browsing reduces the amount of photosynthetic tissues and hence reduces carbon availability within the tree (Bryant et al., 1983; Du Toit et al., 1990). Hence, we expected di‐terpene content to be lower in browsed and clipped trees. However, the control, browsed, and clipped trees had similar di‐terpene contents. This could either be contributed to the high ability for compensation in pine trees (reduction in growth rather than defense) or to the amount of biomass removed was insufficient for a detectable effect on di‐terpene levels. Against our expectation, we failed to find a relationship between sawfly cocoon weight and di‐terpene levels, compared to previous findings of performance being related to needle di‐terpene levels (Björkman et al., 1997; Larsson et al., 1986). The lack of an effect could indicate that the range of di‐terpene levels in individual trees was too small to detect any relationships or the variation was too high. Alternatively, as di‐terpene levels have been found to affect especially early larval survival and potentially development (Larsson et al., 1986) and we added the larvae in their second instar, the effect of di‐terpenes on cocoon weight might have become hard to detect.

The rather weak relationship between insect performance and C:N ratio, and the absence of a relationship with di‐terpene levels, indicates that the observed differences in sawfly performance between the browsed or clipped trees and unbrowsed control trees could be related to additional plant traits. Plant‐mediated effects on insect performance could be either the result of a direct effect through nutritional quality or toxic compounds, or the result of an indirect effect through reduced digestibility (Mattson, 1980). Changes in compounds such as tannins that reduce the amount or the form of available nitrogen are an example of such a candidate plant trait (Feeny, 1968). Tannin levels can be reduced by browsing (Du Toit et al., 1990; Hrabar & Du Toit, 2014), and previous studies demonstrate strong effects on insect pupal mass via tannins in plants (Kaitaniemi, Ruohomäki, Ossipov, Haukioja, & Pihlaja, 1998; Lindroth, Kinney, & Platz, 1993) making them a strong candidate for the potential missing link. Additional candidate traits are the level of other phenolic compounds, which are abundant defensive compounds in pines (Mumm & Hilker, 2006), and have been demonstrated to be affected by browsing (Stolter, 2008) and to affect pupal mass of insect herbivores (Lill & Marquis, 2001). Pasquier‐Barre, Palasse, Goussard, Auger‐Rozenberg, and Géri (2001) showed that phenolic compounds (taxifolin) can decrease performance of the common sawfly, *Diprion pini*, on Scots pine specifically.

Previous studies into effects of within‐species induced response on sawfly cocoon weights have shown variable results. Niemelä et al. (1991) showed no effect on cocoon weight from previous simulated sawfly defoliation (*N. sertifer* and *P. sylvestris*), Lyytikäinen (1994) showed lower cocoon weights on pines with natural defoliation (*N. sertifer* and *P. sylvestris*), and Raffa et al. (1998) found decreased female cocoon weight on previously artificially defoliated pines (*N. lecontei and P. resinosa*). Based on this and results in previous mammal–insect studies (Table 1), we conclude that outcomes could be highly variable and system specific. The positive effect on insect performance presented in our study corresponds to the findings reported by Martinsen et al. (1998) but still there are too few studies to generalize the direction of the effect. As results are variable and point in different directions, elucidating the underlying mechanisms (i.e., plant‐induced responses) could lead to a deeper understanding of indirect effects. Moreover, studying different types of plant systems, such as conifers and deciduous plant, are crucial to bring this field of study forward. In addition, as will be elaborated on below, the time between initiation and receiver response needs to be considered.

In most mammal–insect indirect interactions, the events of herbivory are separated in time (Table 1), which is also the case in this study, creating a potential for legacy effects, that is, plant‐mediated interactions across herbivore generations or species over time (Wurst & Ohgushi, 2015). Studies have shown that the effect on the second herbivore is often larger when herbivores are separated in time, since the plant has had more time to respond (Denno et al., 2000; Erb, Robert, Hibbard, & Turlings, 2011). In addition, many responses diminish over time, and persistence of the response differs, both between responses and systems (Björkman, Dalin, & Ahrné, 2008; Kafle & Wurst, 2018). The effect of initial damage on the receiving herbivore might increase at first but over time decrease, creating a hump‐shaped relationship between time since initial damage and effect on the second herbivore. The amplitude and width of the curve will depend on the measured response in a specific system. We know that the natural browsing damage was inflicted over the duration of winter 2015/16 and the clipping treatment was applied in one day during early spring 2016, resulting in differences in time span between insect feeding and browsing or clipping event. Hence, our results provide starting point for further exploration of the relationship between recovery time of the plant and the magnitude of the trait‐mediated indirect effect. The rather weak effect of browsing on sawfly performance could be due to the relatively long time lag between herbivory events. However, insect performance has been shown to be affected by previous damage occurring even a year earlier (e.g., Neuvonen, Haukioja, & Molarius, 1987). In the light of our, and previous, results, it remains to be elucidated whether the response of the subsequent herbivore depends on the nature of initial herbivore (insect vs. mammal), the specificity of plant species’ defense and the insect's adaptation to it or the timing and extent of herbivory.

## 5 CONCLUSIONS

Our study demonstrates that mammalian herbivory can affect insect performance through trait‐changes in plants. We show that moose browsing could potentially affect fecundity of the European pine sawfly, which could have consequences for multiple ecological processes, especially population dynamics. One important finding of our study is the weak effect of nitrogen on insect performance, opening up possibilities for other compounds as important determinants of performance traits in insects and mediating mechanisms in trait‐mediated interactions between moose and sawflies. We contribute novel insights into the field of indirect interactions, studying a coniferous plant. Additionally, our study system and experimental setup allowed us to investigate indirect effects on insect performance excluding confounding effects of insect density or source. More controlled experimental studies investigating indirect interactions between mammals and insects are needed to deepen the understanding of mechanisms involved and discover consequences of trait‐mediated effects in herbivore communities. To increase general ecological understanding, we advocate more studies of underrepresented systems such as interactions involving taxonomically distant species, not least on conifers.

## CONFLICT OF INTEREST

None declared.

## AUTHOR CONTRIBUTIONS

MN, CB, LE, and MJK conceived and designed the experiment. MN set up the experiment, collected, and analyzed the data. MN wrote the first draft of the manuscript, and CB, MJK, and LE contributed to revisions of the manuscript. AS and JG performed the needle di‐terpenoid analyses.

## Data Availability

Data for this paper are deposited in the Dryad Digital Repository: https://doi.org/10.5061/dryad.8f5847n.
