# Supplementary figures and images for: Trait‐mediated indirect interactions: Moose browsing increases sawfly fecundity through plant‐induced responses
Source: Ecol Evol. 2019 Aug 23;9(18):10615–29. doi: 10.1002/ece3.5581 (PMC6787786; doi:10.1002/ece3.5581)

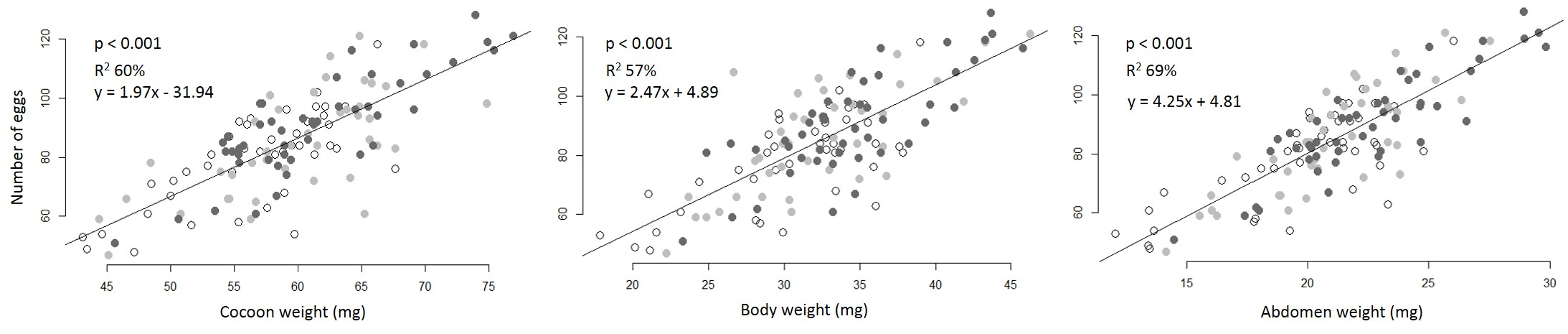

Supplement: Supplementary file 1 [file ECE3-9-10615-s001.png]
