# Supplementary material for: Trait‐mediated indirect interactions: Moose browsing increases sawfly fecundity through plant‐induced responses
Source: Ecol Evol. 2019 Aug 23;9(18):10615–29. doi: 10.1002/ece3.5581 (PMC6787786; doi:10.1002/ece3.5581)
Supplement: Supplementary file 2 [file ECE3-9-10615-s002.docx]

APPENDIX S1.

Method S1. Method for the literature search.

Table S1. Table summarizing previous key research articles on moose browsing – tree response and on pine traits – sawfly performance response.

Fig. S1. Relationship between number of eggs and cocoon weight, body weight and abdomen weight of female sawflies.

Method S1.

*Literature search*

To be able to identify knowledge gaps within the field of trait-mediated indirect interactions involving mammals and insects we conducted a literature search. We conducted the search using two different methods to cover as much of the field as possible, suspecting that there would be little consistency in key words used. We started by using a key-review paper by Ohgushi (2005) as a focal paper for a search. Using a focal paper as a starting point has previously been used successfully to identify studies when there is inconsistencies in the terminology used within the field (Stephens et al. 2013). We identified the citation network for this focal paper (i.e. studies cited in it or studies that have cited it). The final search was conducted on the 15^th^ of November 2018. From the resulting articles, we reviewed the articles that study mammal – insect interactions published before 2005 and thus cited by Ohgushi (2005) (Main document Table 1). Then we applied a four step selection process to narrow down the 244 articles that cite Ohgushi (2005) to the articles focusing on mammal – insect interactions:

1. Filter the studies using the Web of Science filtering function with the search string “mammal” OR “brows*” OR “graz*”
2. Manually remove papers that are not concerned with indirect effects or mammal-insect interactions
3. Remove studies that investigate direct consumptive effects (mammals eating insects)
4. Exclude studies not investigating trait-mediated indirect interactions or studies where it is impossible to separate trait-mediated and density-mediated effects.

Ultimately nine studies remained from the initial 244, which we added to Table 1 resulting in 18 studies on plant mediated indirect interactions between insects and mammals.

Secondly, we did a key word Web of Science search (key word search string described below), identifying 355 papers. The advantage of using multiple key word in the search is that there is a smaller likelihood of missing relevant papers, while the disadvantage is that the search generates many studies that are not relevant but are using similar key words, we for example identified many studies relating to indirect effects of herbivory on plants, and studies in aquatic systems. To deal with this we manually filtered the all papers according to the four following steps:

1. Remove studies wrongly identified, i.e. studies that do not describe indirect interactions between herbivores.
2. Removed studies not using a mammal-insect system
3. Removed studies investigating direct consumptive effects (mammals eating insects)
4. Remove studies where it is impossible to disentangle density-mediated and trait-mediated effects, such as studies investigating plant community effects of browsing/grazing, and following effects on insect herbivores.

This resulted in six additional studies, which were added to Table 1.

Key-word search string in Web of Science:

TS=((”indirect interaction*” OR ”indirect effect*” OR ”trait-mediated interaction*” OR ”plant-mediated interaction*” OR ”interaction modification*” OR ”non-trophic interaction*” OR ”herbivore-herbivore interaction*” OR ”lateral interaction*” OR ”trait-mediated effect*” OR ”plant-mediated effect*” OR ”interaction web*” OR ”subsequent herbivor*” OR ”trophic interaction modification*”) AND (lepidoptera* OR homoptera* OR hemiptera* OR coleoptera* OR thysanoptera* OR hymenoptera* OR diptera* OR orthoptera* OR phasmatodea* OR psocodea* OR fly OR flies OR aphid* OR beetle* OR thrips OR grasshopper* OR caterpillar* OR larvae OR arthropod* OR invertebrate* OR chew* OR suck* OR gall* OR miner* OR mining OR root* OR borer* OR boring OR moth* OR seed* OR folivor* OR insect*) AND (mammal* OR graz* OR brows* OR ”mammalian herbivor*” OR ”large herbivore*”) NOT (marine* OR sea OR ocean OR aquatic OR snail*))

Table S1.

Table S1. Summary of articles studying the effect of browsing on tree chemical responses and the effect of Scots pine traits on sawfly responses. In the browsing – trees part of the table the column ‘tree type’ denotes the type of tree used in the study (when blank a mix of tree types were used) and the column ‘result’ the measured plant response. In the pine – sawfly part of the table the ‘mechanism’ column denotes the plant trait responsible for the effect on the insect and the ‘result sawfly’ column denotes the measure insect response. + signifies an increase, 0 no difference and – a decrease in the measured plant or insect variable.

| Browsing – trees | Study | Tree type | Result |
| --- | --- | --- | --- |
|  | Edenius 1993 | Scots pine | + Nitrogen (non-significant) |
|  | Nykänen & Koricheva 2004 | Evergreens | + Nitrogen |
|  | Danell et al. 1985 | Birch | + Nitrogen (B. pendula, B. pubescens) |
|  | Danell & Bergström 1989 | Birch | – Nitrogen (B. pubescens)  0 N (B. pendula) |
|  | Nykänen & Koricheva 2004 | Deciduous | – Nitrogen |
|  | Nykänen & Koricheva  (damage in general) |  | – terpenes |

| Pine – sawfly | Study | ’Mechanism’ | Result sawfly |
| --- | --- | --- | --- |
|  | Larsson et al. 1986 | + Di-terpenes | – larval development, – survival |
|  | Björkman et al. 1991 | + Nitrogen | + growth, + survival, + cocoon weight |
|  | Björkman et al. 1997 | + Di-terpenes | – pupal weight, – survival |
|  | Niemälä et al. 1991 | (simulated sawfly defoliation) | 0 cocoon weight |
|  | Lyytikäinen 1994 | (natural sawfly defoliation) | – cocoon weight |
|  | Raffa et al. 1998 | (simulated sawfly defoliation) | – cocoon weight |

Figure S1.

*Relationship between number of eggs and weight*

REFERENCES

Björkman, C., Larsson, S., & Bommarco, R. 1997. Oviposition preferences in pine sawflies: a trade-off between larval growth and defence against natural enemies. Oikos, 79(1), 45–52.

Björkman, C., Larsson, S., & Gref, R. 1991. Effects of nitrogen fertilization on pine needle chemistry and sawfly performance. Oecologia, 86(2), 202–209.

Danell, K., & Bergström, R. 1989. Winter browsing by moose on two birch species: impact on food resources. Oikos, 55(1), 11–18.

Danell, K., Huss-Danell, K., & Bergström, R. 1985. Interactions between browsing moose and two species of birch in Sweden. Ecology, 66, 1867–1878.

Edenius, L. 1993. Browsing by moose on Scots pine in relation to plant resource availability. Ecology, 74(8), 2261–2269.

Larsson, S., Ekbom, B., & Björkman, C. 2000. Influence of plant quality on pine sawfly population dynamics. Oikos, 89(3), 440–450.

Lyytikäinen, P. (1994). Effects of natural and artificial defoliations on sawfly performance and foliar chemistry of Scots pine saplings. Annales Zoologici Fennici, 31(3), 307–318.

Niemelä, P., Tuomi, J. and Lojander, T. (1991). Defoliation of the Scots Pine and Performance of Diprionid Sawflies. Journal of Animal Ecology, 60(2), 683–692. doi:10.2307/5305.

Nykänen, H., & Koricheva, J. (2004). Damage-induced changes in woody plants and their effects on insect herbivore performance: a meta-analysis. Oikos, 104(2), 247–268. Raffa, K.F., Krause, S.C., and Reich, P.B. (1998). Long-Term Effects of Defoliation on Red Pine Suitability to Insects Feeding on Diverse Plant Tissues. Ecology, 79(7), 2352–2364. doi:10.2307/176827

Stephens A. E. A. et al. 2013. Strength in numbers? Effects of multiple natural enemy species on plant performance. Proceedings of the Royal Society B, 280, 20122756.

Ohgushi, T. 2005. Indirect interaction webs: Herbivore-induced effects through trait change in plants.Annual Review of Ecology, Evolution, and Systematics, 36, 81–105.

Figure S1. Relationship between number of eggs and a) cocoon weight (mg), b) body weight (mg) and c) abdomen weight (mg). Line represent fitted linear model. Data points represent individual sawflies. Colour of the data points represent the treatment (white = control, light grey = browsed, dark grey = clipped). P-values, R-squared and model equations for the models are presented within each graph.
